# Home is home—Botswana’s return migrant health workers

**DOI:** 10.1371/journal.pone.0206969

**Published:** 2018-11-16

**Authors:** Keneilwe Motlhatlhedi, Oathokwa Nkomazana

**Affiliations:** Faculty of Medicine, University of Botswana, Gaborone, Botswana; National University of Ireland Galway, IRELAND

## Abstract

The shortage of skilled healthcare workers in Sub-Saharan Africa is aggravated by their emigration to high resource countries. There is evidence that a small number of healthcare workers return to their home countries. It is important to understand the factors that influence decisions to return in order to develop appropriate strategies to attract more back. This study sought to investigate the perspectives of healthcare workers who returned to Botswana after working in the diaspora. We conducted semi-structured interviews of 8 healthcare workers. Using the thematic analysis method we developed a thematic index to code the data. The main reasons for returning were family ties and missing home whilst the key reasons for emigration were concerns about the quality of health care, lack of professional progression opportunities and feeling under-valued. Difficulties reintegrating into the Botswana health care system are a potential push factors for those who return. Policies that aim to attract back healthcare workers should address professional progression, reintegration and improvement of the healthcare system.

## Introduction

Sub-Saharan Africa (SSA) has some of the worst health outcomes globally. Almost 50% of the world’s under-5 child and maternal mortality occur in the region [1,2]. Moreover, the region also has a severe shortage of human resources for health with only 10 out of 46 countries in the region having a physician-to-population ratio above the World Health Organisation (WHO) recommended minimum of 2.5 healthcare workers per 1 000 population [3,4]. The exodus of skilled HRH from SSA to developed countries in search of better livelihoods further aggravates this shortage.

Safety and security, low remuneration, poor working environments, professional stagnation and hopes for better lifestyle are among the commonly cited reasons for healthcare worker migration [5–10]. Efforts to curb healthcare worker migration are multi-tiered and include both source country and international strategies. Internationally, bilateral recruitment agreements between source and recipient countries have been developed and an international code of recruitment has been drawn up [4, 6, 8, 9,11,12]. At a local level, donor countries have increased training of both highly skilled and intermediate level healthcare workers and introduced financial and non-financial retention strategies [4, 8].

Migration of healthcare workers from developing countries may have a negative effect, not only on the country, but also on the individual healthcare workers. Some migrant healthcare workers from low-resourced countries are not able to practise in their field of training, resulting in deskilling [6,13,14]. Although the migration of HRH has largely negative effects, some potential benefits have been reported. In the Philippines remittances from healthcare workers are reported as a major benefit of migration, although some authors have noted that this benefit dwindles over time and is not universal [8,14–16]. Furthermore, some healthcare workers do not intend to remit once they migrate [6, 11].

There is insufficient data on return migration of healthcare workers but indications are that migrant healthcare workers are not returning in sufficient numbers to their home countries [4,6,17,18]. Although many migrant healthcare workers report a desire to return, political instability, high crime rates in home countries and the shame of not achieving their migration goals are barriers [5,13,17].

Botswana had its share of loss of health professionals to high-income countries in the face of significant shortages and a dissipating HIV and AIDS epidemic. In 2012, Botswana’s doctor-to-patient ratio was 4:10 000 of the population and at the same time the nurse-to-patient ratio was 42:10 000. These numbers were significantly lower in rural areas: 3 doctors and 26 nurses per 10 000 population compared to urban areas where there were 9 doctors and 77 nurses per 10 000 of the population [19]. Furthermore, only 21% of doctors registered to practise in Botswana were locals. Whereas the majority of nurses in Botswana are trained in the country, other skilled healthcare workers, including doctors, were mostly trained abroad, until 2009 [19]. Over the years many who trained abroad have not returned, contributing to the acute shortage of healthcare workers. Migration also depletes the locally trained healthcare workers with an estimated 7% of Batswana nurses and midwives working in high-income countries [4].

The country established its first medical school and faculty of health sciences in 2009 with the hope to produce adequate numbers of doctors and other allied health professionals to serve its population [20]. Evidence from other countries, has however shown that increasing local training output alone, may not be sufficient to ensure an adequate number of health professionals [4,7]. A multi-faceted approach may be necessary to attract, recruit and retain healthcare workers.

This paper reports on the findings of a study of Batswana (nationals of Botswana) healthcare workers who returned after working in diaspora. The reported study explored factors that influenced the original migration decisions of these healthcare workers and factors that influenced their decision to return. The perspectives of these returned migrants may contribute to developing strategies to curb emigration and increase the return migration of healthcare workers.

## Methods

### Study design

We conducted a qualitative study using, semi-structured in-depth interviews of Batswana healthcare workers who had returned after working in foreign countries.

### Study participants

The interviewees were health professionals who had emigrated from Botswana to work in a foreign country or stayed to work in the country of training after graduation, for at least three months. We excluded all those who worked in foreign countries as part of or concurrent with their training.

### Sampling strategy

The sampling was purposive using Ministry of Health and hospital databases followed by a snowballing strategy to identify more potential participants until there was saturation of themes. Each participant was asked for information on other health workers they knew who had returned after working in another country. The research assistants then contacted the individual telephonically to find out if they met the inclusion criteria and whether they were willing to participate in the study. We aimed to recruit different cadres of health professionals to explore varied experiences and perceptions.

### Data collection

A semi-structured interview guide was used to inquire into: what facilitated migration or the decision to stay in the host country after completion of training, the experiences of working abroad, what enabled the return to Botswana and participants’ experiences of reintegrating into the Botswana health system. The interviews took place in October 2014. Five questions (with prompts) were posed:

1. Can you please tell me the story of your professional journey?
2. Can you please share with me your journey to becoming a health worker in another country?
3. Can you please describe your experience as a migrant health worker?
4. Can you tell me about your journey back to Botswana?
5. What do you perceive to be the benefits of health worker migration to the healthcare system?

The interview guides were piloted with healthcare workers at the University of Botswana clinic. Trained research assistants, all of whom were female, conducted the interviews in English, the official language. The interviews took place in the participants’ workplaces in a quiet room where privacy was assured. The research assistants audio-recorded the interviews and then transcribed them verbatim. Each interview lasted approximately 30 to 40 minutes. ON (Female, MD PhD) who is experienced in qualitative research, then checked all the transcripts. None of the authors had contact with the participants about the research during the data collection. Botswana has a very small health system and ON, who has worked many years in the country, would have known many of the participants and also works with one of them. This was deemed likely to influence responses and hence the use of trained research assistants instead.

### Data analysis

We analysed the data using thematic analysis [21–23]. The transcribed interviews were uploaded on to the ATLAS.ti software. ON and KM read and re-read the transcribed interviews independently. KM then inductively developed codes from the data. ON reviewed the data with the developed codes and after further re-reading both researchers agreed on final codes. Using the code family function on the software similar codes were then grouped into categories based on the identified relationships between the different codes [21]. ON and KM further interrogated the descriptive categories in order to understand experiences of the participants in the context of existing theory on health worker migration. Interpretation of categories led to the themes. Use of the ATLAS.ti software made it easier to identify frequently occurring codes, to link codes to quotations and to create network views which could show the association between codes. The study had intended to enroll ten participants but the enrollment was stopped at nine, mainly because of the difficulty finding participants and also a sense that there were no new ideas being presented.

### Ethical consideration

The University of Botswana Institutional Review Board and the Ministry of Health Research and Development Unit issued permission to conduct the research: Reference No: PPME 13/18/1 V11 (368).

Each participant gave informed written consent to participate in the study. The participants were informed that participation in the study was volitional and that refusal to participate would not lead to any loss of benefits. The participants were also informed that they could choose to exit the study at any point without suffering any consequences.

## Results

Nine participants were enrolled but one of them was excluded in the final analysis as she had gone abroad solely for academic purposes and had returned to the country upon completion of her studies. A total of 12 individuals were approached; one was very reluctant to participate as he was afraid that the information would be used against him, despite assurances of confidentiality; two were never available and after many attempts and appointments they were not pursued further.

The baseline characteristics of the participants are shown in Table 1. Six of the participants are female and half were employed as nurses at the time. The longest period that any respondent had worked outside Botswana was 19 years and the shortest was two years.

**Table 1:** Characteristics of study participants.

	Age	Gender	Marital status	Profession	Current job title	Place of employment	Employment sector	Highest qualification	Country of training	Duration of stay in foreign country (Years)
P1	52	F	M	Nurse	Coordinator		NGO		Botswana	Not stated
P2	40	F	M	Medical Doctor	Medical Officer	City	Not stated	MBChB	UK*	10
P3	50	F	M	Nurse	Gender, HIV and AIDS Advisor	City	NGO	MPH	Botswana, RSA^α^, Australia	2
P4	37	F	M	Medical Doctor		City	Public	Degree		Not stated
P5	39	F	M	Nurse	Nurse Manager		Private	BSc	UK*, Botswana	7
P6	35	M	M	Medical doctor	Doctor	District	Public	MBBS	Trinidad and Tobago, Bahamas	2
P7	47	M	M	Rehabilitation scientist	Rehabilitation Scientist	City	Private	PhD	USA^∞^	23
P9	45	F	S	Nurse anaesthetist	Nurse anaesthetist	District	Public		Botswana	4+

* United Kingdom,

^∞^United states of America,

^α^ Republic of South Africa

**Fig 1:**
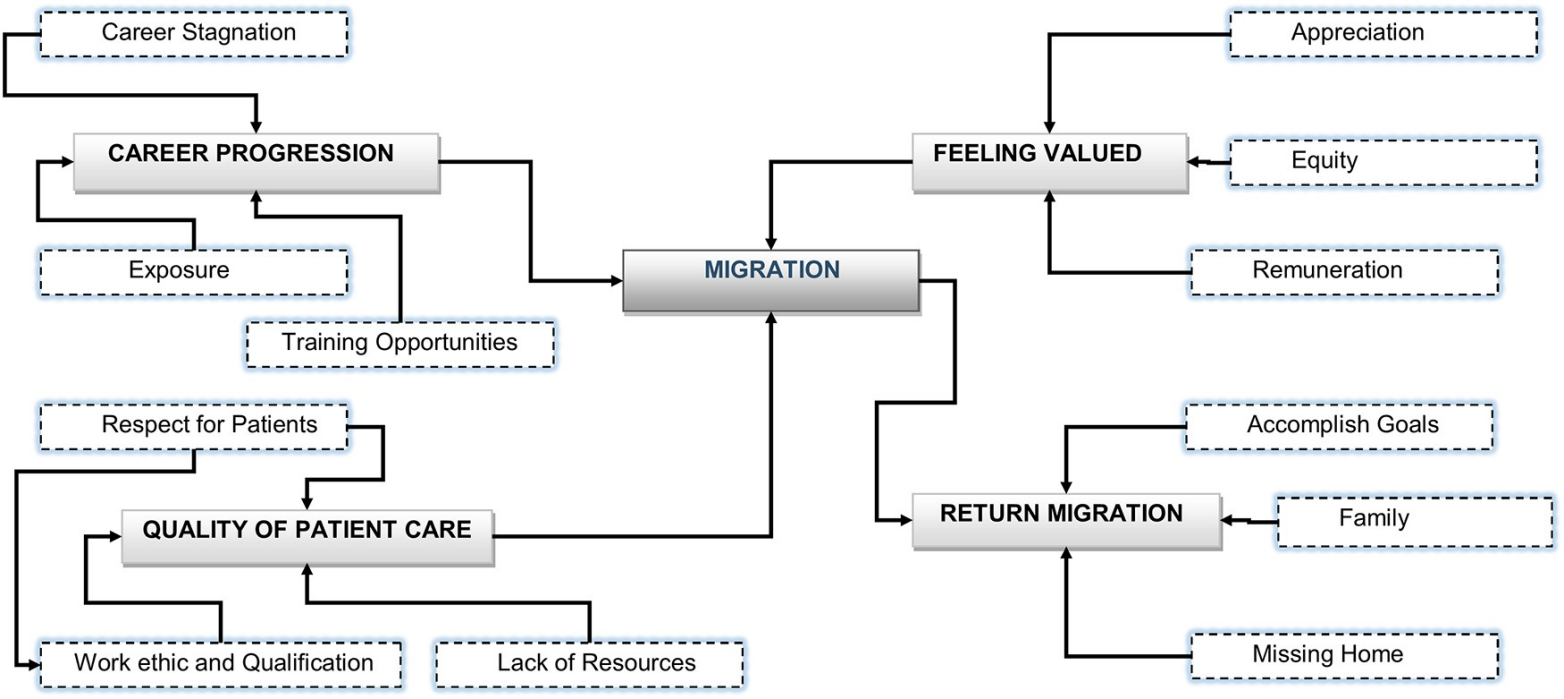
Thematic networks for health worker migration.

The principal themes contributing to migration were push and pull factors [24]. The push factors were mainly health systems related challenges that made practising in Botswana unattractive. Pull factors on the other hand were anticipated benefits of working and living in high income countries. The participants returned home mainly for social reasons. The main reasons for migration are shown in Fig 1.

### Factors influencing emigration

#### Push factors and pull factors

A number of factors were identified as contributing to either push health care workers from Botswana or pull them to foreign countries. The opposites of the factors that pushed health workers from Botswana attracted them to the diaspora. Push factors were mainly health care related and resulted in lack of professional fulfilment. The converse of these factors, however, attracted Batswana health care workers to resource rich countries.

#### Lack of professional fulfilment

Three major issues contributed to lack of professional fulfilment, either experienced or anticipated. These contributors to lack of professional fulfilment were poor quality of patient care, perception of being undervalued and limited career progression opportunities in the home country, Table 2. It is notable that most of the reasons for migration were intrinsic to the public health system, the biggest employer of healthcare workers in the country.

**Table 2:** Main influencers of migration.

Factors intrinsic to the Healthcare system	Reason for migration	Participants
	Poor quality of patient care	2,4,5,6,9
	Perceptions of being undervalued	1,3,4,5,6,7
	Low prospects for career progression	1,2,3,5,7
	Remuneration	1,9,6,3, 7
Factors extrinsic to the health care system	Family	2,4,5

#### Quality of patient care

The factors perceived to influence patient care were availability of resources, qualifications, competencies and work ethics of other healthcare professionals. Unavailability of resources compromised patient outcome leaving the professionals with regrets and highly dissatisfied. This was expressed by one participant as follows:

Every morning you go to work, … you do the best that you can do with the limited resources that you have and then you go home frustrated … at the end of the day you lose patients, you know … you feel like I could have done better if I had 1,2,3.

[P5]

One of the doctors contrast the experience, of how lack of resources affects morale and patient outcome, in Botswana and in diaspora:

When somebody had a heart attack… from what I knew there (country X), you had to rush and try and resuscitate them. … When patients came in….you were able to help them; there were treatments available; you didn’t see people dying from conditions that you knew could be cured. But here it was like oh … you know, we don’t have this… [it is] very frustrated working in such an environment.

[P 2]

Doctors, all of whom trained outside Botswana, found the work ethics in the public health institutions appalling and bordering on being uncaring and disrespectful of patients. As one points out:

… Communication is vital. You have to explain and talk to patients… but here you go … and you say you are going to have this taken off …amputation… they just accept it… because you are the doctor… I feel that patients are not given a chance to ask questions … they are not given options…and it’s very frustrating…”

[P 4]

They contrasted the poor work ethics with their positive experiences in diaspora:

… Professionalism, I just think the work ethic was very different from what I had experienced when I worked here [in Botswana] for 4 months during my elective [in Botswana] … I just feel that when patients came in [in country X] there was that empathy. They were taken seriously.

[P2]

There was also a sense that some of the health workers in the Botswana public health system were not adequately skilled.

People are brave in doing things they are not supposed to do, taking chances … I would say there are not really lots of people now, especially in public hospitals who are properly qualified.

[P9]

I would just say it bluntly on this … you know like people from [xxx country], there is an [xxx] team that is hired by Botswana that I feel, you know, not all of them have good qualifications …

[P4]

#### Career progression opportunities

Professional and career development opportunities were very important sources for professional fulfilment and their scarcity was a big driver for migration or for staying in diaspora. Opportunities for further training in Botswana were rare and even if one managed to get professional training, it did not always translate to career progression. This latter situation especially affected nurses as illustrated below:

… and how many people are in front of me … for me to reach that very post [promotion]…

[P3]

… they will say, in a hospital where there is maybe 400 of you, there are only 2 [training] slots. So you find that the training system, you know, it will take you years before you go [for training]

[P5]

The abundant resources, higher levels of professionalism and opportunities for further training and career progression, in the recipient countries resulted in perceived higher quality patient care which was important for professional fulfilment. Most of the participants also reported working jobs commensurate with the skills and experience.

… I was from a training institution [and] when I got there I was promoted to be a training coordinator *…* matching [my expertise] perfectly.

[P1]

When speaking of opportunities in the recipient country one participant notes

how far you go professionally is to a large degree determined by how hard … how hard you are willing to work…if you are willing to work hard then financially you will be very stable…[something that allowed] professional development.

[P7]

#### Perception of being undervalued

Almost all the participants felt no appreciation for their work by the Ministry of Health—the original employer of most of the participants before migration. The perception emanated from lack of rewards or recognition for their work and skills as exemplified below:

You work but um … I guess there is no appreciation of what we do to a certain extent.

So eventually some get discouraged and say “oh I better go … there my work is appreciated better …

[P6]

Nurses were particularly aggrieved by the lack of recognition of further training and skills acquisition. One of the participants actually, who had completed advanced professional training in Australia, left to go to UK because lack of recognition and promotion.

I did not enjoy it because [government] considered that going to school is an advantage [and not a guarantee] that from there you will be automatically promoted .… So it meant that you are now stagnant … whereas you have come back with some advanced training.

[P1]

Disparities in salaries within and between professions was highly resented as it was seen as demonstrating the low value afforded them. Doctors resented the differences in salaries favouring a group recruited by government through a special arrangement with a foreign recruitment company. This was particularly begrudged as many of the recruited specialists were regarded as less qualified than the locals. One of the doctors passionately retorted:

The salaries you are giving expatriates and foreigners … if you were to give them to Batswana I am telling you, (you) would be able to retain a lot of Batswana.

[P4]

Other allied health professions were unhappy at the time because of disparities in salaries between the different cadres. Physical therapists, for instance resented being paid lower salaries than pharmacists and laboratory technologists.

As one points out:

You will see the pharmacists for example, I am using the pharmacists [as an example] … I don’t even know if this has been resolved …many of us felt that the pharmacists were treated in a way that is much better.

[P 7]

This led to a mass exodus of physiotherapists in the 1990s.

Quiet a number left, in fact I think it started the year before until around 1993–1994…

[P7]

Remunerations were also a significant pull factor as many hoped to afford a better lifestyle, save money for the education of the children and acquisition of property back home. These factors encouraged those in Botswana to migrate and acted as a stay factor for those already in diaspora, either as students or workers. As an illustration:

… the money was …is always part of it because the salaries over there are more than here … and when you are there your life would be different from when you are here because things are cheap.

[P5]

Even remuneration here it’s quite low by any standard. You compare ours with Namibia and South Africa. Ours is very low; so that has a bearing [on migration]…

[P6]

Table 2 below illustrates the main factors influencing migration of the healthcare workers from Botswana.

### Factors influencing return to Botswana

The prime reasons people returned to Botswana were social. These included missing home and family challenges, Table 3. It is interesting to note that the main reasons people return to work in Botswana were extrinsic to the health system.

**Table 3:** Reasons and facilitators of return migration.

Factors extrinsic to the healthcare system	Reason for return	Participants who stated this reason
	Missing home	1,4,5,6,9
	Family	1,2,6,7,9
	Achieved goals	1, 5,9
	Racism	1,4,5
	weather / satisfaction from serving your own people	1, 4/6
Factors intrinsic to the Healthcare system	Ease of employment	3,5,7

Missing home was a prevailing sentiment among the participants and most never intended to remain permanently in diaspora. The following quotes illustrate this:

Home is home I was missing my whole family, my extended family and I just wanted to be home and I am glad I am home.

[P4]

You know home will always be home …my family was there with me, my kids and my husband, we were together so we were comfortable but we were still missing home.

[P5]

They always say home is home… no matter what, so I feel that was… eventually I think all of us eventually do come home it is a matter of when.

[P6]

Family was also an important influencer on return migration. Some participants expressed that child-care was expensive in the recipient countries whilst others had left their children behind.

I felt so … you know, that pain such that … I wondered that my child had grown this big when I left him so young … so 100 per cent my reason for coming back was because of the little … child.

[P1]

I suppose the other influences especially when you have children: child care issues they are just much more complicated in terms of money, hours, laws and regulations… again you thought, at home I can just get a helper very easily.

[P2]

In addition, family responsibilities were one of the commonest reasons people decided to return to Botswana. As these participants commented:

My wife was here, she was nursing (our baby) I ended up saying let me just do temporary work… we were not yet settling but we ended up settling.

[P6]

I had my elderly mother that I really thought, eish, let me go back to my mother.

[P9]

My parents are aging, both my parents and my wife’s parents, in-laws, and (I) said no we can contribute to make things a bit easier for them during their last days on earth so to speak. So it was more of a social reason.

[P7]

#### Accomplishing personal goals

Some participants had gone abroad with specific targets and returned home once these had been achieved. However, many spent more time abroad than they had originally planned:

To go and make a living … as being there I would afford to send money home to Botswana, to build houses I wanted … you know … there would come a stage after houses and cars and my children having gone to schools and feeling satisfied that I would then come back.

[P1]

… because at the end of the day you have a reason why you went there and what you wanted to achieve. And after achieving what I wanted to achieve then I came back home.

[P5]

I like the neonatal area and I did that and I acquired the skills [completed training] … I was like now I am ready to run this department in my country. Then after that I decided to come back and here I am.

[P5]

Although participants in this study had experienced some discrimination whilst working abroad, this experience did not play a major role in their decision to come home. This is illustrated in the quote below

“… you are not in your own country of course … it’s not going to be easy …People will always remind you where you come from, they will ask you “why did you come here?” there is that element of racism that exists … but you have to develop a thick skin… they will always feel like you have come to take their things…”

[P5]

### Reintegration into Botswana’s health system

The ease of reintegration into the Botswana health system depended on whether one was joining the private or the public health sector. Most of those who joined the private health sector had jobs lined up for them before coming back into the country and there was therefore minimal frustration:

… to me I wouldn’t say it was difficult … I think it was just straightforward because I got the job while I was still in the UK … I am not working for government I work for private …

[P5]

Even coming [back] here [to Botswana], I was headhunted.

[P3]

Another participant had been made aware of opportunities with the newly founded medical school.

because when I was here my friends said why don’t you check what the school of medicine may offer… despite the fact that I knew that it will be a huge pay cut.

[P7]

Those who joined Government experienced delays in being employed once back home, which was very challenging. One participant even entertained thoughts of returning to the UK:

But… it wasn’t easy. There was lot of frustration and lot of waiting and being tossed to and fro … no proper … channels … It was a bit frustrating. At one point I actually thought of going back …

[P4]

I was told ‘there is no job’… I don’t take ‘no’ from anybody, you don’t just tell me no. So I went to the office of the minister.

[P9]

One participant explained that reintegration had been easier because she had maintained work relations with Botswana

Because I have continued participating in the work that Botswana was doing … that’s what I think was an advantage for me.

[P3]

The primary drivers of return migration for participants in this study are indicated in Table 3 below.

### Benefits of migration

All the participants felt that migration was potentially beneficial to the country. The work ethic and the professionalism they acquired were important competencies they believed could be transferred.

They had also gained clinical skills and knowledge that other health professionals in Botswana would benefit from:

I am looking forward now for doctors trained here [in Botswana] to go and work outside … because once they come back they [would have seen] even conditions that are not here … nurses who are coming back have seen more advanced equipment that are used…

[P3]

… For me, when I came back with the skills that I had gained there…I think that would be beneficial to Batswana…I had worked in a big renal transplant dialysis unit… the knowledge and the culture of appraising the evidence.

[P2]

## Discussion

To the best of our knowledge this is the first study to investigate the perspectives of returned Batswana healthcare workers. The research explored perceptions of different health worker cadres who returned to Botswana after working abroad for varying lengths of time.

Health system related factors were the main push factors for migration. Factors contributing to migration in this study are mainly intrinsic to the health system. The major reasons Botswana health workers emigrated or remained in diaspora after completing training were lack of professional fulfilment and perception of being undervalued by the employer, who in most cases was the Ministry of Health. Although the government health facilities in Botswana have relatively good infrastructure and are equipped with basic health equipment, there are reported shortages in medications and equipment as well as staff shortages; all of which negatively impact service delivery [25]. This limitation of resources was reported by participants in this study and associated with poor patient outcome which many of the health professionals found distressing. This emotional stress and strain due to inadequate disastrous patient outcome was also reported by Blacklock et al in their meta-ethnographic synthesis of migration decisions of African health workers and trainees [26]. Evidence from Ghana also strongly links health worker motivation to quality improvement activities and patient safety [7]. This challenge is addressed by the WHO strategy to increase human resources for health by improving infrastructure and supplies, which will create a more functional health care environment [4].

Lack of opportunities for professional and career development reported in this study is a well-recognised push factor for migration [21,24,27]. The WHO report of 2006 urges health systems to promote lifelong learning which will address this need for professional development [4].

Feeling undervalued and unfairly treated was an important contributor to migration in this study. This perception of being undervalued and of injustice in recognition and remuneration, in the Botswana public health system, has been reported by other studies in the country [28,29]. Injustice as a push factor has also been reported in Zimbabwe where government’s agreement with Cuba imported hundreds of Cuban doctors and nurses who were much better remunerated than locals [10]. The WHO urges countries to ensure appropriate remunerations and adequate information and communication, this strategy may address feelings of injustice as experienced by the participants [4].

Although there is much written about the factors influencing the intentions of migrant health care workers to return home, there is not much written about the factors influencing those healthcare workers who eventually did return. In this study factors extrinsic to the health care system were the principal influencers of return migration: Homesickness, family and having accomplishing economic and professional goals were cited as the primary reasons for returning home. The role of that family played in the migration decisions of healthcare workers may be more pronounced in this study since most participants were married.

These reasons for going back to one’s home country were also expressed in other migration studies. Family ties and missing home country lifestyle and culture, for instance, were some of the main factors that influenced the return intentions of Sub-Saharan health care workers working in Europe [17,30]. Family ties are a mixed motivator for migration, serving as both a pull factor to the home country and a barrier to returning, although for our group of participants, it was one of the main reasons for return.

African migrant healthcare workers in Europe also reported a desire to return to their home countries so that their children would get to know their motherland and its language and culture [17]. Many of these have nevertheless reported that they face insurmountable barriers to return to home countries. These included war, personal safety, economic meltdown, “institutional crisis” and political instability [5,17]. It may be easier for Botswana’s healthcare workers to return if the health systems weaknesses are addressed as issues of security and economy have never been raised as either push or stay factors. Whereas caring for younger children may be easier in source countries where there is family support, older school going children may be deemed better off in high income countries with perceived higher quality education. This suggests that parents of school going children may stay longer in recipient countries to allow their children to complete their studies, whilst those with younger children may opt to return home where childcare is deemed more affordable and family support feasible.

Exploring ways to further enhance this connectedness with one’s home country may improve the chances of migrant health care worker return. The World Health report 2006 emphasises the need for health care systems to accommodate the needs of healthcare workers by subsidising childcare, creating working conditions that are family friendly and facilitating the return of workers who have been out of the system [4]. This recognition of the importance of social factors in the movement of healthcare workers is echoed by the findings of our study.

The report further advocates for institutions to facilitate links between migrants and local (source country) institutions. The return of migrant healthcare workers may also be encouraged by maintaining contacts with source country. Knowing of opportunities in one’s home country as well as an awareness of the need for a particular skills set may encourage migrants to return. This is supported by the report of one respondent in this current study who had returned home because she was head hunted by the home country, having maintained professional contacts with them.

Keeping abreast of what is happening in their home countries allows healthcare workers to plan for and adjust to the differences in healthcare settings. An awareness of what is going on in one’s healthcare systems also invokes a sense of duty to one’s country or hope that things are improving [4,17,30]. However, sense of duty does not always translate to an actual permanent return. Some healthcare workers in the diaspora plan to visit their home countries regularly to contribute to their home health system whilst still working abroad [14,30,31].

As in our study the attainment of professional targets was also found to be a major contributing factor to the decision to return home for migrant healthcare workers in several studies [14,30,31]. This is not surprising since opportunities for further training are a frequently identified pull factor in human resource for health migration studies. Professional and personal development are recurring themes in the emigration and immigration decisions of healthcare workers. The skills and knowledge gained whilst working in high income countries can greatly benefit health systems of lower income countries such as Botswana [29,30,31]. This potential benefit of migration is only possible if migrant healthcare workers return to their home countries, even if for short periods.

In our study, those who chose to work in the public health system faced great difficulty finding jobs and integrating into the health system. This can potentially deter those in diaspora who may have intentions to return to the country or encourage them to selectively look for employment in the private sector. Delay in employment may even encourage re-migration, as one of the participants hinted. It is therefore critical to make the transition as smooth as possible and not to seem to be punishing the health workers for working abroad.

It is clear from this study that health system related factors play a major role in the migration of Botswana health professionals and a seemingly minor role in their return. This is corroborated by other studies in the country [28,29]. Consequently strategies to stem migration should address health system factors broadly and not only focus on remuneration, which is often the case. The perception of injustice requires special attention as it is not widely reported in migration literature. Further studies are also required to understand this phenomenon fully and also find effective context specific inclusive strategies to address these perceptions.

Factors influencing the movement of healthcare workers are many and complex hence multifaceted and context specific approaches to reducing outward migration and increasing return migration are required [15,32–34]. Improving the healthcare system in general is necessary to maintain job satisfaction and increase retention [35,36] This can be done by ensuring that resources are available and clear progression policies are developed and implemented in a fair and transparent manner [4,30]. Additionally, expediting the postgraduate training and continuing professional development of healthcare professionals is likely to increase the number of healthcare professionals who stay in their country, as many who leave are in search of higher education [9, 10,15,23,27].

This study showed that some healthcare workers return once they have attained the desired migration goals. Inevitably there will always be some healthcare workers who emigrate. Creating a database of all healthcare professionals may provide insight into who is more likely to move and where they go. Understanding the movement patterns of healthcare workers may help inform future policy [29]. These policies may include allowing healthcare workers to go on sabbatical leave or short-term employment in higher-resource countries.

### Study limitations

A limitation of the study is the use of a purposive sampling strategy, as some informants with different experiences may have been missed. The study sample was small and may not reflect the spectrum of views held by healthcare workers who return to Botswana. There was no triangulation although the data on reasons for migration was similar to the perceptions of different stakeholders (healthcare workers, policy makers and members of the community) in an earlier study conducted by O.N. et al. [28]

## Conclusion

Botswana, like most low-resource countries, is faced with a shortage of human resource for health which is exacerbated by migration. There is also an undocumented number of health workers who return after working abroad. Factors intrinsic to the health system seem to be the major drivers of migration while social issues tend to drive return migration. An improved understanding of the contribution of these different factors in migration decision-making processes of healthcare workers is important. It is also important to appreciate that these healthcare workers obtain valuable experience which can significantly improve the healthcare systems of their home countries with proper facilitation. Often positives such as these are neglected by conceptual models that strive to explain the migrant situation. Since migration is invariably going to continue to some degree, building strategies that encourage return may help to turn potential brain and financial drain to brain gain.

Future studies are necessary to explore the conceptual understanding of the migrant phenomenon and quantify the actual numbers of Batswana who return from the diaspora. It is also important to find out if those who return actually remain in the country and assess their impact on the health system.
